# Characterisation of *Dermanyssus gallinae* glutathione *S-*transferases and their potential as acaricide detoxification proteins

**DOI:** 10.1186/s13071-015-0960-9

**Published:** 2015-06-26

**Authors:** Kathryn Bartley, Harry W. Wright, Robert S. Bull, John F. Huntley, Alasdair J. Nisbet

**Affiliations:** Moredun Research Institute, International Research Centre, Pentlands Science Park, Bush Loan, Penicuik, Midlothian, EH26 0PZ UK

**Keywords:** *Dermanyssus gallinae*, Poultry red mite, Glutathione *S*-transferase, Acaricide resistance

## Abstract

**Background:**

Glutathione *S-*transferases (GSTs) facilitate detoxification of drugs by catalysing the conjugation of the reduced glutathione (GSH) to electrophilic xenobiotic substrates and therefore have a function in multi-drug resistance. As a result, knowledge of GSTs can inform both drug resistance in, and novel interventions for, the control of endo- and ectoparasite species. Acaricide resistance and the need for novel control methods are both pressing needs for *Dermanyssus gallinae,* a highly economically important haematophagous ectoparasite of poultry.

**Methods:**

A transcriptomic database representing *D. gallinae* was examined and 11 contig sequences were identified with GST BlastX identities. The transcripts represented by 3 contigs, designated *Deg*-*GST*-*1*, −*2* and −*3*, were fully sequenced and further characterized by phylogenetic analysis. Recombinant versions of *Deg*-GST-1, −2 and −3 (r*Deg*-GST) were enzymically active and acaricide-binding properties of the r*Deg*-GSTs were established by evaluating the ability of selected acaricides to inhibit the enzymatic activity of r*Deg*-GSTs.

**Results:**

6 of the identified GSTs belonged to the mu class, followed by 3 kappa, 1 omega and 1 delta class molecules. *Deg*-GST-1 and −3 clearly partitioned with orthologous mu class GSTs and *Deg*-GST-2 partitioned with delta class GSTs. Phoxim, permethrin and abamectin significantly inhibited r*Deg*-GST-1 activity by 56, 35 and 17 % respectively. Phoxim also inhibited r*Deg*-2-GST (14.8 %) and r*Deg*-GST-3 (20.6 %) activities.

**Conclusions:**

*Deg*-GSTs may have important roles in the detoxification of pesticides and, with the increased occurrence of acaricide resistance in this species worldwide, *Deg*-GSTs are attractive targets for novel interventions.

## Background

The haematophagous poultry red mite [*Dermanyssus gallinae* (De Geer, 1778)] is the most economically important parasite affecting commercial egg production facilities worldwide, causing an estimated annual production loss of €130 million in Europe alone [1, 2]. Infestation levels are estimated to range from a “normal” 50,000 mites per bird to over 500,000 mites per bird in severe infestations [3]. The impact on bird health and behaviour can be severe and manifests as agitation and pecking, weight loss and anaemia; with production losses attributed to increase bird mortality, a decrease in egg quality and output, higher feed-conversion rate and mite control costs [2, 4, 5]. In addition, *D. gallinae* is as a potential vector for a number of diseases, some of which may also impact on human health; for example, Erisipelas and Salmonella spp. [6–11].

Chemical-based mite control is the usual approach employed in commercial premises and several organophosphate, pyrethroid, spinosyn and carbamate acaricides are licensed for use in the EU [5]. Resistance to organophosphates, pyrethroids, carbamates and dichlorodiphenyltrichloroethane (DDT) has been identified in red mite populations across the EU [12–17]. However, the underlying mechanism(s) involved in acaricide detoxification and resistance in *D. gallinae* remain unknown. In addition to active site mutation, the development of acaricide resistance in other pest species may be attributed to the activities of detoxifying enzymes including members of the glutathione *S*-transferase (GST), cytochromeP450 (CYP450), and carboxylesterase (CBE) superfamilies and may be multi-factorial in nature [18]. The CYP450 and CBEs function in phase I of detoxification (modification) by catalyzing the addition of polar groups to xenobiotics. Phase II (conjugation) most often involves the GST-catalysed conjugation of reduced glutathione (GSH) to the electrophilic xenobiotic [19]. The glutathione conjugate acts as a flag, targeting the xenobiotic to the membrane transporters, whereupon it is excreted from the cell in phase III of the detoxification process [20].

GSTs belong to a large superfamily of versatile proteins with important functions in detoxification, amino acid catabolism, signal modulation, transportation [21]. Cytosolic and mitochondrial GSTs are classified in several canonical classes (mu, kappa, alpha, pi, theta, sigma, zeta, omega, lambda, beta, tau, delta and epsilon) dependent on the structure of class-specific active site motifs, active site residues and binding affinities [22, 23].

GSTs are highly important in the detoxification processes in haematophagous parasites, particularly in the elimination of toxic haemoglobin breakdown products [24–26]. GSTs have also been identified as major allergens in several parasite and pest species (e.g. scabies and house dust mites, parasitic nematodes and cockroaches), that provoke pathophysiology associated with allergy [27–31]. In addition, secreted GSTs have been implicated in immune-evasion of the nitric oxide response in helminths [32]. Given their pivotal role in detoxification, drug resistance and host-parasite interaction, GSTs have been the focus of novel control strategies against a number of ecto- and endoparasites [33–38].

The identification of *D. gallinae* GSTs (*Deg*-GSTs) will therefore lead to a deeper understanding of acaricide resistance in this species and may lead to novel targeted control methods. We have previously produced a *D. gallinae* transcriptomic database [39] and, in the present study, we identify the repertoire of GSTs in *D. gallinae* by data-mining the database with GST search terms and investigate the interactions of 3 selected *Deg*-GSTs with acaricides commonly used in the poultry sector to characterise the potential for involvement of the *Deg*-GSTs in acaricide detoxification.

## Methods

### Generation of *D. gallinae* transcriptome dataset

Mixed stage and gender *D. gallinae* mites collected by scraping aggregated mites from the enriched-cage system of a commercial egg-laying unit in Scotland, UK. Mites were allowed to migrate away from the detritus and were then snap frozen in liquid nitrogen within 12 h of collection. Total RNA was purified using Trizol reagent (Life Technologies) as previously described Wright [39]. Roche 454 sequencing was performed (Edinburgh Genomics, University of Edinburgh) following the manufacturer’s protocol [40, 41]. The raw read data were assembled using Newbler (version 2.3) into contig sequences and putative biological functions inferred by homology searching against the NCBI nr database (19 July 2013 release) using BLASTx [42]. Where possible, the *D. gallinae* trancriptomic dataset was further annotated with Gene Ontology (GO) terms, Enzyme Commission (EC) codes and InterProScan (IPS) identities using the Blast2GO pipeline [43–45].

### Amplification and sequencing of selected *D. gallinae* glutathione *S*-transferases (*Deg*-*GSTs*)

The complete coding sequences of selected *Deg*-*GST*s were obtained using rapid amplification of cDNA ends (RACE) using the SMART™ RACE cDNA amplification kit (Clontech) according to the manufacturer’s touchdown protocol and where necessary or, where full length sequence was already represented in the assembled contigs, by direct amplification from cDNA by PCR using the Advantage® 2 PCR Kit (Clontech). Sequences of cloned cDNAs representing three GSTs, *Deg*-*GST*-*1*, −*2* and −*3* were determined following cloning in pGEM® T-Easy plasmid (Promega). Homology searching of translated *Deg*-GST sequences against the UniProtKB database (release 2013/06) was performed using blastp. Additionally, Signal P v4.0 [46] was used to identify putative signal sequences and ScanProsite [47] to identify functional domains and protein signature sequences in the translated *Deg*-GST sequences. A selection of proteins with highest homology to the *Deg*-GSTs were aligned with the translated *Deg*-GST along with arthropod and eukaryotic GSTs belonging to selected GST classes, using ClustalX v2.0.11 [48]. The constituent proteins were manually truncated at C and N terminus by up to 29 amino acids in order to eliminate regions of extensive gapping and poor alignment. The truncated alignment was imported into the TOPALi v2.5 [49] and the Whelan & Goldman model (WAG) amino acid substitution model was applied to the alignment to calculate distances. A phylogenetic tree was constructed using the MrBayes Bayesian tree method [50] using default parameters. The integrity of each partition or clade (clade credibility) was assessed using posterior probabilities.

### Expression, purification and activity of recombinant *Deg*-GSTs (r*Deg*-GSTs)

The complete coding sequences of the *Deg*-*GST*s were subcloned into the pETSUMO champion plasmid vector (Invitrogen, primer sequences and conditions available on request from authors). For recombinant protein expression, purified pETSUMO-*Deg*-GST plasmid DNAs were transformed into *Escherichia coli* BL21-CodonPlus® (DE3)-RIL competent cells (Stratagene) and protein expression, purification and quantification performed as described previously [51]. Purified r*Deg*-GSTs were used for electrophoresis on NuPAGE® Bi*s*-Tris 4-12 % gels under reducing conditions and stained with SimplyBlue™ (Invitrogen). To confirm identity of the r*Deg*-GSTs, the single visible bands were excised, destained and subjected to reductive alkylation using DTT and iodoacetamide. Gel pieces were digested overnight at 37 °C in trypsin and digests analysed on an Ultraflex II MALDI-ToF-ToF mass spectrometer (Bruker Daltonics). The masses obtained were used for database searching with the MASCOT search engine using Swis*s*-Prot and local databases with a 50 ppm mass tolerance window. Significant matches from the Peptide Mass Fingerprint data were confirmed by MS/MS analysis using the search criteria above and an MS/MS tolerance window of 0.5 Da.

The ability of the purified r*Deg*-GSTs to catalyse the conjugation of reduced glutathione (GSH) to 1-chloro-2,4-dinitrobenzene (CDNB) was assessed and quantified by measuring the increase in absorbance at 340 nm (A_340nm_) over time as described in Lee et al. [52]. Briefly, 20 μl volumes containing defined quantities of the r*Deg*-GSTs in Tris/NaCl buffer (10 mM Tris, 0.5 M NaCl, pH 7.4) were placed, in triplicate, into the test wells of a 96-well microtitre plate and the equivalent volume of Tris/NaCl buffer into the control wells. 100 μl CDNB at serial concentration range of 0 mM to 1.0 mM (in 0.2 mM increments) were added in substrate buffer (100 mM potassium dihydrogen phosphate, 1 mM EDTA, pH 6.5; 2 mM GSH), to all wells. The A_340nm_ was measured immediately and every minute thereafter for 20 min (ELx808IU microplate reader, BIO-TEK instruments, Inc.) while the reaction proceeded in a linear phase. The mean A_340nm_ of the triplicate control wells was subtracted from the mean of the triplicate tests wells at the different CDNB concentrations to adjust for spontaneous hydrolysis of CDNB. The specific activities (μmol/min/mg protein) of r*Deg*-GSTs at the different CDNB concentrations were calculated using the formula:

$$ \frac{\left(\mathrm{A}\mathrm{t}2\hbox{-} \mathrm{A}\mathrm{t}1\right)\times 1000}{\mathrm{A}\upvarepsilon \times \left(\mathrm{t}2\hbox{-} \mathrm{t}1\right)\times \mathrm{b}\times \mathrm{m}}\times \mathrm{Vtot} $$

where the At1 and At2 are the mean adjusted A_340nm_ at the initial and final time points; Δε the molar extinction coefficient of CDNB (Δε =9.6). t2 – t1 is the time (in minutes) between At2 and At1; b is the path length of the spectrophotometer (cm = 0.286); m is the quantity of r*Deg*-GST per well (in mg) and Vtot is the total volume per well (litres).

### Acaricide inhibition assays

5 mM stocks of the acaricides: spinosad, permethrin, phoxim and abamectin (PESTANAL® analytical standards, Sigma) and 50 mM 4-nitrobenzyl chloride (NBC) were prepared in ethanol. Acaricide, NBC (+ve control) or ethanol (no treatment control) was placed (2.4 μl) into triplicate wells of a 96-well microtitre plate and mixed with 20 μl r*Deg*-GST [containing defined quantities of r*Deg*-GSTs in Tris/NaCl buffer (10 mM Tris, 0.5 M NaCl, pH 7.4)]. The plate was incubated for 10 mins at R.T. with agitation prior to the addition of 100 μl per well of 1 mM CDNB substrate in substrate buffer. GST activity was measured as detailed above and adjusted for background absorbance of the different acaricides at A_340nm_. The assay was repeated on 3 separate occasions and the mean enzyme activities analysed using a one-way ANOVA followed by pairwise comparisons using Tukey’s post-hoc analysis to assess significance.

## Results

### Identification and characterisation of *Dermanyssus gallinae* GST transcripts

The annotated *D. gallinae* transcriptome containing 13,363 contiguous sequences (contigs) [39] was interrogated using glutathione *S*-transferase keywords and 11 contigs were identified with GST terms. These 11 contigs ranged from 208 to 1030 bp in length and, based on BLASTx descriptions, 6 were putatively identified as mu class, 3 as mitochondrial kappa class, 1 omega and 1 delta class.

The 3 longest contigs with the highest number of constituent sequences and top hit BLASTx e values were selected for further study. The contig forming *Deg*-*GST*-*1* was 1016 bp in length, comprised of 58 overlapping sequences and had a 48 % identity (over 237 amino acid residues, E = 8e-72) with a putative mu-1 like GST from the highly related predatory mite species *Metaseiulus occidentalis* (accession number XP_003747409). The 1030 bp contig representing *Deg*-*GST*-*2* comprised of 33 overlapping sequences and possessed 77 % identity (over 208 amino acid residues, 1.0E-77) with a putative *M. occidentalis* GST isoform C-like GST (accession number XP_003743487). The contig representing *Deg*-*GST* −3 was formed from 5 overlapping sequences, had a total length of 399 bp and a 76 % identity (over 66 amino acid residues, 2.0E-25) with a putative *M. occidentalis* mu-1 like GST (accession number XP_003742682).

The contigs representing *Deg*-*GST*-*1* and −*2* were both predicted to contain the full coding sequence (CDS) of their respective GSTs (Accession numbers KR337505 and KR337506) and this was confirmed using PCR amplification. The contig representing *Deg*-*GST*-*3* encoded a partial open reading frame (ORF) and the full length sequence (Accession Number KR337507) was obtained using RACE. The ORF of *Deg*-*GST*-*1* was 735 bp in length, encoding a 244 amino-acid (aa) protein; the ORF of *Deg*-*GST*-*2* was 633 bp encoding a 210 aa protein and the ORF of *Deg*-*GST*-*3* was 669 bp encoding a 222 aa protein.

**Fig. 1:**
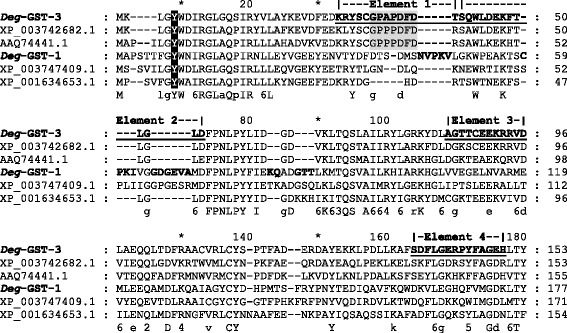
Conservation of glutathione *S*-transferase (GST) mu class elements and protein signature motifs in the N-terminal region of two GSTs derived from *Dermanyssus gallinae*. The N-terminal regions of two *D. gallinae* GST proteins (*Deg*-GST1 and *Deg*-GST-3) were aligned using the ClustalX algorithm with their two closest BLASTp matches: *Metaseiulus occidentalis* (XP_003742682.1 and XP_003747409.1), *Nematostella vectensis* (XP_001634653.1) and *Haemaphysalis longicornis* (AAQ74441.1). The signature motifs of mu class GSTs are annotated as follows: the conserved tyrosine active site residue (black shading), the four mu class elements (PR01267, bold and underlined) and the mu-loop structure (grey shading). The amino acid insertions conserved in *Deg*-GST-1 and XP_003747409.1 are in bold

All 3 *Deg*-GSTs possessed GST N-terminal (IPRO04045) and GST C -terminal (IPR004046 and IPRO10987) motifs as well as N-terminal thioredoxin-like fold (IPRO12336) and GST C-terminal chloride channel (IPRO17933) motifs which are typical of all classes of GSTs. Database scanning identified the four elements of the mu class signature sequence motif (PRINTS: PRO1267) in *Deg*-GST-3, however these elements were not observed in the other putative mu class GST, *Deg*-GST-1 (Fig. 1). Likewise, a loop structure characteristic of mu class GSTs was highly conserved in *Deg*-GST-3 (G^34^PAGPFD^40^), but poorly conserved in *Deg*-GST-1. The divergence of *Deg*-GST-1 protein sequence from typical mu class GST was also apparent from several short sequence insertions that are not present in any of the other mu class aligned sequences, except *M. occidentalis* where these insertions are partially conserved. Despite the apparent divergence of the two putative mu class *Deg*-GST (−1 and −3), both matched the PDBeMotif panther PTHR11571:SF3 subfamily comprised of functional mu class GSTs. The tyrosine amino acid responsible for GST activation in the G-site of mu, phi, alpha and sigma GST classes is conserved in *Deg*-GST-1 (Tyr^9^) and *Deg*-GST-3 (Tyr^5^). Activation of the G-site in delta, theta and zeta class GSTs occurs with a serine rather than tyrosine [53] and the Ser^9^ of *Deg*-GST-2 is conserved supporting the putative class designation of *Deg*-GST-2 as theta or delta class.

**Fig. 2:**
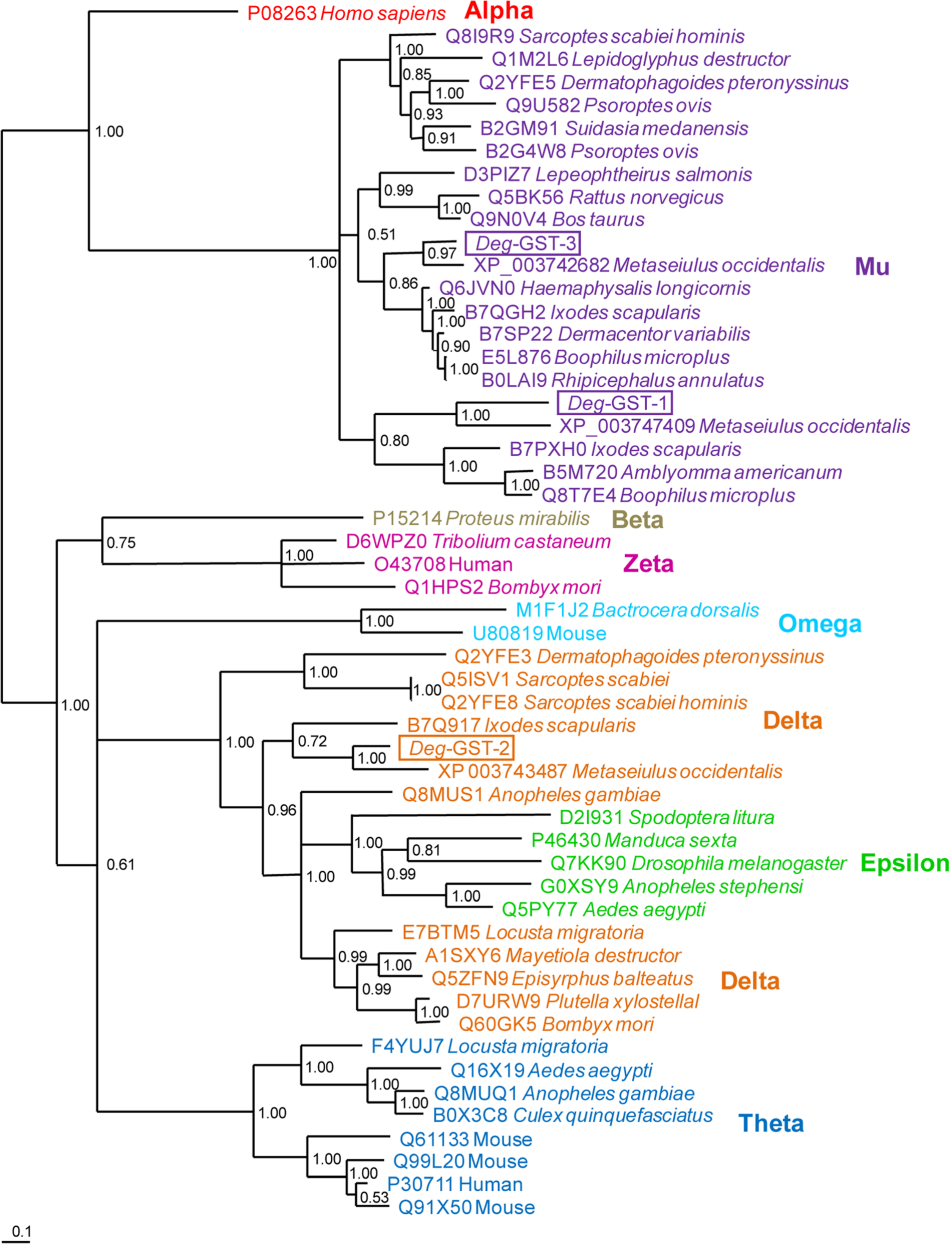
Phylogentic analysis of three glutathione *S*-transferase (*Deg*-GST) protein sequences derived from *Dermanyssus gallinae*. Three *Deg*-GST protein sequences (*Deg*-GST −1, −2 and −3) were aligned using the ClustalX algorthim along with a selection of homologous and protypal proteins belonging to the selected classes of GSTs: alpha, mu, beta, zeta, delta, omega epsilon and theta. The constituent proteins were manually truncated at the termini to eliminate regions of poor alignment. The phylogenetic tree was determined using the default parameters of MrBayes Baysian tree method with the Whelan & Goldman model (WAG) amino acid substitution model. The accession number and species of each protein is presented. The 3 *Deg*-GSTs are boxed. The clade credibility of each partition was assessed using posterior probabilities and is presented at each node

A phylogenetic analysis of the 3 *Deg*-GSTs and 50 representative GSTs from vertebrates and invertebrates, encompassing alpha, mu, beta, zeta, omega, delta, epsilon and theta classes is presented in Fig. 2. Both *Deg*-GST-1 and *Deg*-GST-3 partitioned with the mu class GST clade. Within the mu clade, the GSTs grouped into 3 defined subclades, supported by a strong clade credibility (CC = 1.0). The first mu subclade contains members of the superorder Acariformes (e.g. *Sarcoptes* spp. and *Psoroptes* spp. mites). The second mu subclade partitioned further into 3 clusters containing mammalian- and crustacean-derived GSTs, those from the Mesostigmatid mites and the Ixodid tick species. The third subclade also partitioned into 2 clusters containing the Mesostigmatid mites and Ixodida ticks respectively. Both putative mu class *Deg*-GST s (−1 and −3) partitioned mostly closely with the Mesostigmatid mite *M. occidentalis*.

*Deg*-GST-2 partitioned into a large clade containing delta and epsilon GSTs. The representative epsilon GST species formed a cluster discrete from the delta GSTs which, in turn, formed one cluster containing mainly acarine-derived sequences and one cluster containing exclusively insect-derived sequences. As with the *Deg*-GST mu class GSTs, the *Deg*-GST-2 also partitioned most closely with *M. occidentalis*.

### *Deg*-GST activity

**Fig. 3:**
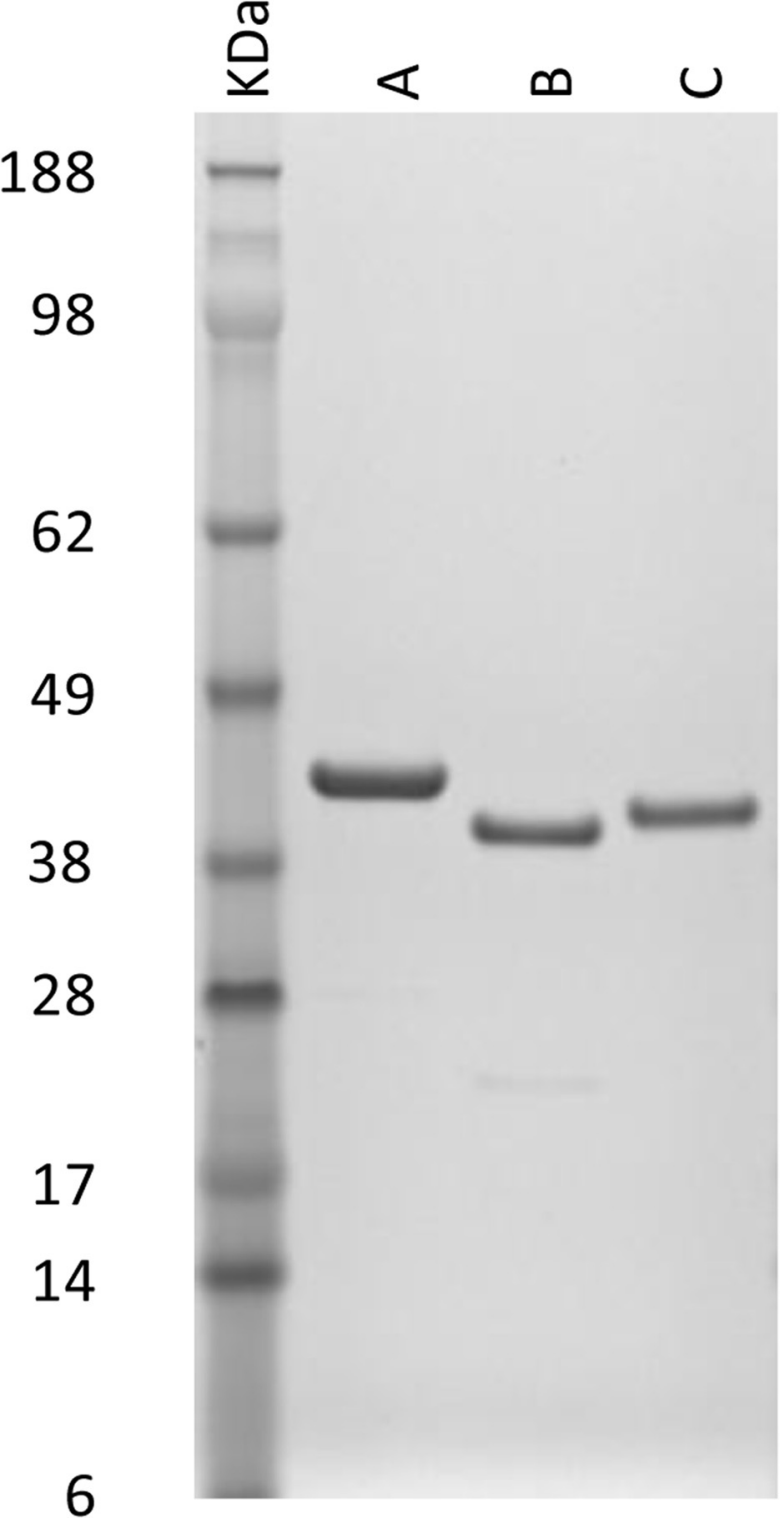
Purity of recombinant versions of three *Dermanyssus gallinae* GST proteins (r*Deg*-GST −1, −2 and −3). r*Deg*-GST-1 (lane A), r*Deg*-GST-2 (lane B) and r*Deg*-GST-3 (lane C) were produced as fusion proteins with N-terminal His-tags and SUMO peptides then affinity purified through a HisTRAP column(GE healthcare) and the imidazole removed by dialysis against 10 mM Tris, 0.5 M NaCl, pH 7.4. Four μg of each r*Deg*-GSTs were denatured and electrophoresed on a 4–12 % Bis-Tris Novex gel (Invitrogen). Proteins were visualized with SimplyBlue™ SafeStain and molecular masses of the r*Deg*-GSTs estimated by comparison with SeeBlue® Plus2 pre-stained protein standards (Invitrogen)

All three r*Deg*-GST- SUMO-fusion proteins were successfully expressed in *E. coli* as soluble recombinant proteins. Following affinity purification and dialysis to remove imidazole, the r*Deg*-GSTs were analysed by SDS-PAGE and the inferred molecular masses of the purified proteins were consistent with the calculated masses of the respective fusion proteins (Fig. 3). The identities of the purified r*Deg*-GST proteins were confirmed using MALDI-ToF mass spectrometry.

**Fig. 4:**
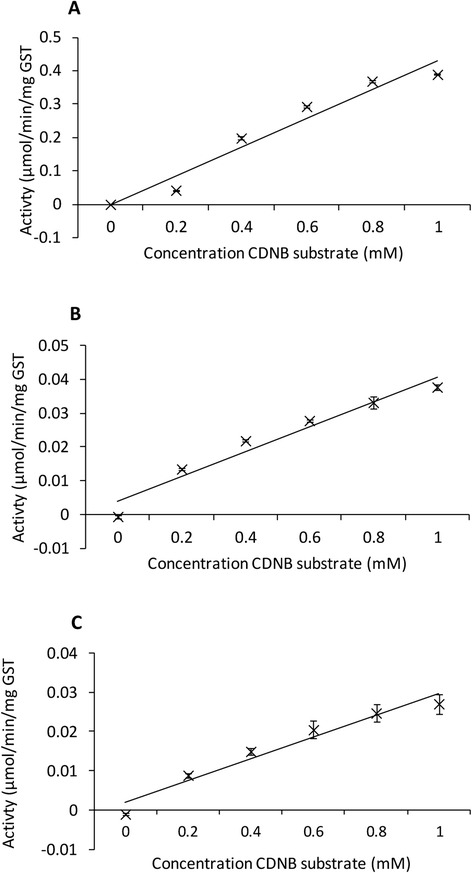
Enzymatic activity of three recombinant *Dermanyssus gallinae* GSTs proteins (r*Deg*-GSTs). The enzymatic activity of the purified r*Deg*-GST −1, −2 and −3 (panels **a**, **b** and **c** respectively) was determined over a 20 min period by measuring the increase in absorbance at 340 nm (A_340nm_) resulting from the GST-driven processing of the colorimetric 1-chloro-2,4-dinitrobenzene (CDNB) substrate present at a range of concentrations (0 to 1 mM). All assays were performed in triplicate with a constant concentration of 2 mM reduced glutathionine (GSH). The A_340nm_ was adjusted for spontaneous substrate decay prior to calculating the mean specific activity (μmol/min/mg GST). The mean specific activity at the different CDNB concentrations is shown (± SEM, *n* = 3)

All three r*Deg*-GSTs were enzymically-active molecules capable of catalyzing the conjugation of GSH to CDNB substrate. The activity of each enzyme (μmol CDNB conjugated with GSH/min/mg GST) was calculated and plotted against CDNB concentration (Fig. 4). A linear relationship was observed between the r*Deg*-GST and substrate concentrations within this range. r*Deg*-GST-1 possessed the highest specific activity. Comparison of the activity of the three r*Deg*-GSTs with CDNB substrate at a concentration of 0.4 mM showed that r*Deg*-GST-1 was 18.3 times more active than r*Deg*-GST-2 and 26.8 times more active than r*Deg*-GST-3.

### Interaction of *Deg*-GSTs with acaricides

The ability of several acaricides to interact with the r*Deg*-GSTs and thus inhibit their ability to catalyze the conjugation of GSH to CDNB was demonstrated using the acaricide inhibition assay (Table 1). The inhibitory effect of acaricide interaction with the r*Deg*-GSTs was quantified by measuring the decrease in absorbance at 340 nm (A_340nm_). The lowest possible concentrations of r*Deg*-GSTs (given in Table 1), that allowed a constant GST activity over a 20 min period, in the presence of 1 mM CDNB and 2 mM GSH, were used in the assays in order to maximise the detection of inhibition. The conjugation of reduced glutathione with CDNB by all 3 r*Deg*-GSTs was significantly reduced [confidence intervals (CI) ≥ 95 %] by the presence of NBC, which served as a competitive-inhibition positive control. Phoxim was the only acaricide to significantly reduce (CI ≥ 90 %) the activity of all 3 r*Deg*-GSTs. Phoxim also produced the greatest reduction in r*Deg*-GST activity: a reduction of 56 % with r*Deg*-GST-1. Conversely, spinosad was the only acaricide that had no significant effect on the activity of any of the r*Deg*-GSTs tested. Permethrin and abamectin also did not have any significant (CI ≥ 90 %) effect on the activity of r*Deg*-GST-2 and −3, but showed a 35 and 17 % reduction in r*Deg*-GST-1 activity respectively.

**Table 1:** The effect of selected acaricides on the ability of r*Deg*-GSTs to catalyse the conjugation of glutathione to the colorimetric substrate CDNB

Treatment	Concentration	% reduction in *Deg*-GST activity ± SEM		
		r*Deg*-GST-1 (0.3 μg GST/well)	r*Deg*-GST-2 (1.5 μg GST/well)	r*Deg*-GST-3 (1.5 μg GST/well)
NBC	1.0 mM	50.84 ± 1.4^b^	18.1 ± 1.7^b^	33.3 ± 2.1^b^
Spinosad	0.1 mM	−17.46 ± 3.6	1.7 ± 5.5	−18.8 ± 5.7
Permethrin	0.1 mM	35.39 ± 0.9^b^	0.5 ± 3.0	−0.30 ± 0.3
Phoxim	0.1 mM	56.38 ± 3.0^b^	14.8 ± 1.5^a^	20.56 ± 2.5^a^
Abamectin	0.1 mM	17.31 ± 2.1^a^	4.3 ± 4.2	−0.57 ± 9.6
No treatment	NA	0.0 ± 0.0	0.0 ± 0.0	0.0 ± 0.0

r*Deg*-GSTs were incubated together with appropriate defined concentrations of acaricides, 4-nitrobenzyl chloride (NBC) or ethanol diluent (as a no-treatment control) for 10 mins at RT prior to the addition of 1.0 mM CDNB substrate. The final concentrations of acaricides and NBT used in these assay were selected on the basis of their solubility and inhibitory effect in several similar GST inhibition assays [68–70, 85]. The absorbance at 340 nm (A_340nm_) was measured immediately (background absorbance of the treatments) and again at 20 mins. The assay was repeated 3 times. The A_340nm_ values at 20 mins were adjusted for background absorbance and the percentage reduction in *Deg*-GST activity, compared to the no treatment control, calculated and is presented along with ± SEM. Differences in the activities of each r*Deg*-GST in the presence or absence of each acaricide were assessed using a one-way ANOVA followed by pairwise comparison using Tukey’s post-hoc analysis

^a^Significant 90 % simultaneous confidence intervals using Tukey post-hoc test of ANOVA data

^b^Significant 95 % simultaneous confidence intervals using Tukey post-hoc test of ANOVA data

## Discussion

In this study we have identified the repertoire of *Deg*-GSTs in a transcriptomic dataset and selected and 2 mu class and one delta class *Deg*-GST for further characterisation. We have shown that several commonly used poultry acaricides have the potential to affect the function of recombinant versions of the selected *Deg*-GSTs. Control of *Dermanyssus gallinae* is becoming increasingly difficult due to the emergence of acaricide resistance coupled with the legislated withdrawal of previously effective acaricides [5], for example, the organophosphate fenitrothion is now banned in the UK [15]. In addition, the application of acaricides, sometime illegally, has resulted in acaricide residues in eggs, meat and feed at concentrations greater than the maximum residue limits in the EU and elsewhere [54–56]. Our understanding of the mechanisms involved in acaricide resistance in *D. gallinae* are in their infancy, though organophosphate resistance has clearly been linked with increased acetylcholinesterase (AChE) activity [57]. Molecular studies underpinning our fundamental understanding of poultry red mite biology were also lacking until very recently, when large quantities of transcriptomic data were generated [39, 58, 59]. In the transcriptomic dataset interrogated here, the most frequent BLASTx term associated with known detoxification pathways was gluthionine *S*-transferase. Of the 11 contigs with associated GST terms, 6 were putatively ascribed to the cytosolic mu class. The bias towards mu class GSTs in *D. gallinae* was also evident in the *D. gallinae* transcriptomic data published by Schichts et al. [59], where of the 32 isotigs identified with associated GST terms, 22 were putatively classified as mu. The high representation of mu class GSTs is in agreement with genomic analyses of another haematophagous parasitic acarine species, *Ixodes scapularis*, where *in silico* genome analysis revealed that mu was the predominant GST class followed by delta, epsilon, omega, zeta and kappa [60]. This is in contrast to the insect species, *Tribolium castaneum* and *Anopheles gambiae*, which lack the mu class entirely; with the mu class replaced by an expanded delta class [61, 62]. Delta class GSTs were canonically defined as insect-specific [19, 63], however, delta class GSTs have now been described here from *D. gallinae* and previously from several other species of the Acari (e.g. *Sarcoptes scabiei*, *I. scapularis* [60, 64] and *P. ovis* [S Burgess, personal communication]. Epsilon and zeta class *Deg*-GSTs were not identified in the red mite transcriptomic data sets, which was unexpected because they have been identified in other acarine species genomes [60] and epsilon and delta classes are often expanded in other haematophagous arthropods [61]. However, the apparent absence of these classes from the red mite transcriptomic databases may be due to very low levels of gene expression and thus representation in acarine transcriptomic datasets: for example, the *P. ovis* transcriptome (12,160 contig/isotig assembly) has only 2 zeta class GST isotigs and no epsilon class GSTs (personal communication S Burgess). Transcripts representing sigma class GSTs were also absent from the *D. gallinae* transcriptomic data, which is in agreement with other Acari species e.g. the *I. scapularis* genome [60] and the *P. ovis* transcriptome [S. Burgess personal communication].

Phylogenetic analyses of the 3 selected *Deg*-GSTs consistently placed the *Deg*-GSTs within the *Parasitiformes* superorder. Mites belonging to the *Acariformes* superorder (e.g. the Astigmid mite species *P. ovis* and *S. scabiei*) partitioned in a separate clade. In all cases, *Deg*-GSTs partitioned most closely with *M. occidentalis*, which is unsurprising as both species belong to the suborder *Dermanyssina* and *M. occidentalis* is the most closely taxonomically related species included in the phylogenetic analysis.

All 3 r*Deg*-GSTs exhibited activity with CDNB; a ubiquitous substrate for all GST classes except theta class [19], however, r*Deg*-GST-1 was 18 and 26 times more active than r*Deg*-GST-2 and r*Deg*-GST-3 respectively. The variation in *Deg*-GST activity is likely to be the result of underlying primary sequence differences affecting the affinity for CDNB, as even single amino acid substitutions in or near to the active sites of GSTs have been shown to dramatically alter CDNB binding ability [65]. Variation in GST structure arising from allelic variation also impacts on detoxification *in vivo* and such allelic variation has been associated with altered binding specificity of GSTs to DDT in the mosquito *Anopheles gambiae* [66]. The generation of alleleic variants provides a mechanism whereby differential pesticide-binding specificities can arise and result in pesticide-tolerant phenotypes. However, no evidence for GST-allelic variation associated with acaricide resistance in Acarid populations has been reported to date.

Here, we tested the ability of a range of commonly-used poultry sector acaricides to interact with r*Deg*-GSTs and the greatest reduction in GST activity was measured when r*Deg*-GST-1 activity was inhibited in the presence of the organophosphate compound phoxim. Phoxim was also the only acaricide that produced a statistically significant reduction in activity in all 3 r*Deg*-GSTs indicating that the 3 r*Deg*-GSTs tested here may play a role in phoxim detoxification. Whether the binding between the r*Deg*-GSTs and phoxim is a functional interaction resulting in a competitive inhibition of GST activity with the CDNB substrate (i.e. phoxim acts as a reducible substrate for GST-mediated GSH conjugation) or whether phoxim is non-competitive inhibitor (i.e. phoxim binds to, and directly inhibits the function of the r*Deg*-GSTs with conjugation to GSH) is not yet clear. Phoxim, and other organophosphates, irreversibly binds to acetylcholinesterase (AChE), thus preventing the AChE-mediated breakdown of acetylcholine (ACh) and the natural cessation of nervous impulses [67]. The resulting overstimulation of the acarine nervous system eventually leads to paralysis and death. In addition to the interaction of phoxim with *Deg*-GSTs shown here, several other commercially-available organophosphates have been shown to interact *in vitro* with GSTs derived from other acarine species [68–70], indicating a potential role for GSTs in the detoxification or inactivation of organophosphate pesticides in the Acari. A five-fold increase in the expression of a GST transcript in the tick *Rhipicephalus microplus* following exposure to coumaphos [71, 72] and a ten-fold increase in GST activity in organophosphate-resistant *Tetranychus urticae* mite extract [73] adds weight to the argument that over-expression of GSTs may play an important role in organophosphate detoxification and resistance in the Acari.

The inhibitory effect of permethrin on r*Deg*-GST activity was limited to r*Deg*-GST-1, where a 35 % decrease in activity was observed in the acaricide inhibition assay. The interaction between GSTs and permethrin and the inhibition of GST function has also been identified in other tick and mite species using similar acaricide inhibition assays [68–70]. In addition, the increase in GST transcription in response to sublethal pyrethroid treatments in several mite species [74–76] and the increased susceptibility of *Rhipicephalus sanguineus sensu* lato ticks to the effects of permethrin when an abundant mu class GST is inhibited via RNAi [77] indicates an important role of GSTs in the detoxification processes of pyrethroids in the Acari. The exact nature of the interaction of permethrin with GSTs and their role in detoxification remains unclear: the interaction between the insect *Tenebrio molitor* GST and decamethrin involved direct binding to the enzyme’s active site, but did not result in conjugation of the pesticide with GSH, suggesting sequestration of the decamethrin, thereby preventing it’s interaction with the target ligand [78]. Mutations in and/or increased expression of the effector molecules of detoxification pathways are often associated with pesticide resistance and pyrethroid resistance mediated by esterase and CYP450 over-expression as well as SNPs in the voltage-sensitive sodium channel have all been documented in the Acari [72, 74, 79–81].

The increase in GST gene expression and GST activity in response to treatment with pyrthroids (as discussed above) suggests that increased GST activity is also likely to be part of a multi-factorial detoxification system involved in pyrethroid resistance in the Acari. There have been numerous reports of pyrethroid resistance in *D. gallinae* [12, 13, 15, 17] and the potential existence of point mutations in GSTs associated with pyrethroid resistance, allowing increased affinity for pesticides, has been explored in other mites, but thus far none have been identified [82]. The mechanism by which GSTs function in pyrethroid resistance may therefore revolve around increased expression of the enzymes rather than alterations in their structure; thus an increase in the transcription of 6 GSTs in the citrus red mite (*Panonychus citri*) following exposure to a sublethal dose of fenpropathrin [76]. In *S. scabiei*, an increase in GST activity in permethrin-resistant mites when compared to permethrin-sensitive mites is associated with mite survival following exposure to permethrin [74, 75]. Furthermore, when pyrethroid-resistant scabies mites were treated with the GST inhibitor and acaricide synergist diethyl malate (DEM), the resistant phenotype was abolished and sensitivity to permethrin restored [75].

Spinosad (Elector®) has recently been registered for use in poultry pest control and has shown to be very effective against all motile stages of *D. gallinae* [83, 84]. No significant change in activity of the 3 r*Deg*-GSTs was observed following incubation with spinosad suggesting that these particular *Deg*-GSTs do not have high affinity for this acaricide. Abamectin is a macrocyclic lactone-containing pesticide, which was approved for commercial *D. gallinae* control in the UK in 2012 [5]. The effect of abamectin on r*Deg*-GST activity was limited to 17 % reduction in r*Deg*-GST-1 activity, and, although spinosad and abamectin do not appear to interact to any great extent with the r*Deg*-GSTs tested here; this does not preclude the possibility of other *Deg*-GSTs functioning in the detoxification of these compounds.

## Conclusions

We have shown that mu and delta class GSTs are present in *D. gallinae* and interact with phoxim, permethrin and abamectin acaricide compounds and they therefore may have important roles in the detoxification of several classes of pesticides. To further characterise the role(s) of *Deg*-GSTs in detoxification and resistance, it would be constructive to perform *in vivo* studies with mites quantifying the *Deg*-GST activity levels and gene expression profiles following exposure to acaricides in mite populations with and without defined acaricide exposure and resistance. Identifying *Deg*-GSTs involved in the detoxification of acaricides may allow the development of targeted treatment design to disrupt detoxification and hinder the mites’ ability to survive acaricide treatment. Selectively targeting and disrupting *Deg*-GST function could therefore potentially work in synergy with existing acaricides to increase their efficacy and prolong their use in the field by overcoming resistance.
